# Transformed but Not Cured: The Ethics of Describing Gene‐Editing Therapy for Sickle Cell Disease

**DOI:** 10.1002/hast.70033

**Published:** 2026-05-06

**Authors:** Jada Wiggleton‐Little, Shameka Poetry Thomas, Kristin Walters, Consuela Albright

**Keywords:** sickle cell disease, SCD, gene editing, doctor‐patient communication, lived experience, bioethics, pain, cure

## Abstract

*In December 2023, the U.S. Food and Drug Administration approved gene‐editing therapies as sickle cell disease treatments. Such approvals for gene‐editing not only mark radical scientific innovations for populations living with sickle cell disease (SCD) across the United States but also generate an expectation of a potential cure—the end or eradication of an illness and its effects. This essay, however, cautions against framing gene‐editing therapy as a “cure” for SCD. Our argument illustrates that, even if gene editing is proven to permanently normalize the hematologic function of the body, there are other painful aspects of SCD that gene editing is unable to transform. Scientific researchers and health care practitioners could benefit from further bioethical consideration of the effects of using curative language with regards to SCD. The curative framing can easily generate misunderstandings in patient‐provider communication and elicit unrealistic expectations. Raising awareness about the importance of how gene‐editing therapies for SCD are described and about the need to discuss their limitations can prevent further harm*.

## Essay

In December 2023, the U.S. Food and Drug Administration approved two cell‐based gene therapies, Casgevy and Lyfgenia, for treatment of sickle cell disease. News outlets have said that gene therapy for SCD provides a “new life”^1^ or a “new hope for sickle cell disease cure” and have referred to those who received the therapy as being “cured.”^2^ SCD is a life‐threatening genetic blood disorder that has as a defining symptom recurring painful and debilitating vaso‐occlusive crisis, or sickle cell pain crisis, across the life course. For more than a century since the disease was first identified, research on it has been widely underfunded, and clinical trials for SCD treatment have been slow to develop. Thus, the FDA's approval of the gene‐editing therapies Casgevy and Lyfgenia not only marks radical scientific innovation for populations living with SCD across the United States but also generates a promise to cure SCD. However, little attention has been paid to how curative language can be detrimental for this patient population. What does it even really mean to “cure” SCD?

In this essay, we argue that, as individuals navigate sickle cell treatment and gene‐editing procedures, it is crucial to be cautious about using curative language to discuss the benefits of gene editing for SCD. As demonstrated in public media, there is an inclination to refer to gene editing as a “cure,” and this tendency can unintentionally be perpetuated by clinicians and manufacturers of the gene therapy. Instead, we emphasize the importance of using language that clearly communicates the limits of gene‐editing therapy for the disease. To be clear, we are not against gene therapy. We are merely against the use of curative language to describe it. Such language misrepresents the lived experiences of patients who receive these treatment options. While referring, as Lyfgenia's manufacturer does, to gene therapy for SCD as “transformative therapy” is better at managing patients’ expectation, this language, too, masks the significant limits of that transformation, as gene therapy does not free people from all burdens of the disease. We recommend engaging with patients with SCD and their caregivers to find language that clearly articulates the fact that gene editing can transform the blood but cannot cure organ damage, that gene editing can transform pain caused by vaso‐occlusive crises but cannot eradicate ongoing mental trauma and social suffering accumulated from the lived experience of SCD, and that gene editing can transform reproductive health but cannot cure sickle cell transmission between mother and fetus and does not address the lack of access to fertility preservation.

## The Limitations of Curative Framing

Until the FDA's recent approval of gene editing, bone marrow and stem cell transplants were considered the only curative options for patients with SCD. However, less than 15 percent of patients with SCD in the United States have an appropriately matched donor.^3^

In cell‐based gene therapy, patients’ hematopoietic stem cells are modified with CRISPR–Cas9 technology and then infused back into the patient, where they generate healthy, or nonsickled, red blood cells. As a result, gene therapy can alleviate vaso‐occlusive crises, halt the progression of organ damage caused by SCD, and decrease the financial and physical burden of ongoing SCD management.^4^ Moreover, unlike other treatment options for SCD, gene‐editing therapy avoids the need for a matched donor and avoids the risks of rejection and graft‐versus‐host disease.

Even if gene‐editing therapies are successful at permanently eradicating sickled cells from the blood of those with SCD—which research has yet to prove—it is misleading to frame these and similar one‐time therapies as “cures” for SCD. Pharmaceutical companies emphasize a reduction in vaso‐occlusive crises, or sickle cell crises, as the curative benefit of gene therapy. “Cure” suggests the normalization of the body, the obtaining of a normal life, or a positive change in one's illness identity.^5^ Thus, curative language sells a promise to alleviate debilitation at every level of the mind and body conjunction. Often, a “cure” is understood as a restoration of health, with the eradication of an illness and its effects being equated to the restoration or normalization of a body's function.^6^

Vertex Pharmaceuticals Incorporated, manufacturer of Casgevy, describes this product as a “one‐time therapy that may help people twelve years and older with SCD and frequent vaso‐occlusive crises (VOCs) live severe VOC‐free.”^7^ The effectiveness of this treatment is also cashed out in terms of vaso‐occlusive crises, with the website stating that, “in the clinical study, 93.5% (29 out of 31 people) did not have a severe VOC for at least 12 months in a row after receiving Casgevy” and that “the average length of time without a severe VOC was 22.2 months (measured as a median amount).”^8^ There is no mention as to whether the clinical study participants continued to experience chronic pain more broadly. This focus on vaso‐occlusive crises captures but one dimension of sickle cell‐related pain, perhaps excluding other important aspects of patients’ lived experiences. There has been little research on patients’ attitudes and perspectives toward gene therapy following the FDA approval of its use for the treatment of SCD, but, in one study, over 80 percent of SCD patients and caregivers reported that “the potential benefits of improved quality of life, longer life expectancy, and benefit to others with SCD in the future would” sway “their decision to pursue gene therapy.”^9^ Although these anticipated benefits are related to a reduction in vaso‐occlusive crises, these findings indicate that the study's participants believed that gene therapy promises more than that.

While Bluebird Bio (now Genetix Biotherapeutics) refers to their gene therapy treatment, Lyfgenia, as a “transformative therapy” for SCD, how they invoke this language of transformation can also encourage patients to hold unrealistic expectations of their lives after treatment. A transformative therapy is a therapy that provides a transformative, meaningful change in a patient's life compared to existing alternatives. The idea that Lyfgenia will transform a patient's life is further reinforced by the slogan on the patient‐facing website for the treatment: “Someday life with sickle cell disease could be different.”^10^ SCD can be an all‐consuming illness. Although gene therapy transforms the blood and reduces the expected number of vaso‐occlusive crises, Lyfgenia's marketing can create a false sense of hope in SCD patients given the few aspects of their lives (and their pain) that are within this treatment's scope of transformation.

## Transformed Blood but Not Transformed Pain

The eradication of sickled blood cells does not equate to the eradication of sickle cell‐related pain. Sickle cell pain is often reduced to the experience of sickle cell crisis, an acute hematologic problem, which ignores the physiological, neurological, and social aspects that play a role in sickle cell pain.^11^

Gene therapy may transform the blood, but the pain of bodily damage and opioid dependence caused by a history of repeated acute vaso‐occlusive events can nevertheless persist. We can see this by considering the limitations of bone marrow transplant, which has been a common treatment option for SCD patients for decades. The persistence of these other kinds of pain is seen in the case of CJ, a forty‐one‐year‐old man with SCD who opted to undergo a bone marrow transplant from a sibling donor.^12^ Prior to the transplant, CJ would receive weekly opioid prescriptions and would visit the rapid‐access clinic two to three times per week. After the transplant, CJ spent two months in the hospital due to complications like mucositis (painful sores in the mouth and intestinal tract) and graft‐versus‐host disease. Although CJ's bloodwork showed no evidence of sickle cell in his blood, he continued to experience chronic pain that was aggravated by mental anguish following the transplant. Despite having been “cured” of his SCD, CJ was still requesting weekly opioid prescriptions and visits to the rapid‐access clinic. How could it be that CJ was still experiencing chronic pain, and for how long should his pain have been treated like that of a patient with SCD?

Many adults with SCD experience chronic daily pain despite diagnostic tests showing no indicators of a vaso‐occlusive event. A diary study showed that adults with SCD reported pain on 56 percent of days even though crisis‐related pain could account for only 12 percent.^13^ Unlike the acute pain associated with a sickle cell crisis, chronic SCD pain can have many underlying causes. Prolonged or repeated nociceptive pain events can cause hyperalgesia, or neural changes that can enhance one's sensitivity to noxious stimuli or heighten one's awareness of and attention to painful sensations.^14^ Chronic exposure to high doses of opioids—an appropriate treatment for acute vaso‐occlusive crises—can also lead to chronic SCD pain via opioid‐induced hyperalgesia and cyclic opioid withdrawal. Moreover, chronic SCD pain, like other chronic pain conditions, is associated with anxiety and emotional distress, which can exacerbate pain.^15^

In sum, describing gene‐editing therapy with curative language would keep hidden the neuropsychological and social factors of sickle cell pain, factors that are common and well acknowledged in other chronic pain syndromes. Many health care practitioners are hesitant to use terms like “cure” or “cured,” instead opting for language like “transformative therapy,” “treatment,” “in remission,” or “no evidence of disease.”^16^

## Social Suffering and Persistent Stigmatization

Patients might think that to be “cured” of SCD is to be “cured” from the stigma and discrimination that was brought on by the illness. But gene editing cannot eradicate all the social suffering associated with SCD. Patients with SCD are more likely to be stigmatized as “drug seekers,” and their pain testimonies are more likely to be met with doubt or disbelief. In one study, 63 percent of nurses surveyed said that many patients with SCD are addicted to opioids, when the estimated prevalence of opioid addiction in patients with SCD is no higher than the estimated 10 percent for the general population who receives an opioid prescription.^17^ This myth that those with SCD are more likely to become addicts stems from the fact that SCD is racialized as a “Black disease” and, in the United States, there is a dominant social logic that conflates Blackness with criminality.^18^ Moreover, Black patients’ reports of pain are more likely to be discredited by clinicians than are white patients’.^19^ Such prejudicial beliefs result in what philosopher Miranda Fricker refers to as a “testimonial injustice,” or the unfair downgrading of a person's credibility due to prejudice against the patient's, or speaker's, group membership.^20^

Given that gene editing transforms the blood without transforming the lived experience of pain, it is quite possible that a patient whose SCD‐related pain was already doubted may experience even worse dismissal after they are “cured.” Such was the case for Genesis Jones, who, following a stem cell transplant in 2020, still struggled with chronic back and leg pain.^21^ She noted that physicians would sometimes turn her away because her blood tests no longer indicated that she had SCD. The absence of any indication of a vaso‐occlusive event may encourage a physician to interpret a report of sickle cell‐related pain as unintelligible or doubtful.

Prior research of stigma related to hepatitis C suggests that undergoing gene therapy will not necessarily eradicate the stigma of presumed drug use associated with SCD. No longer having the viral infection associated with hepatitis C did not necessarily end the social effects of such a heavily stigmatized disease. While those living with hepatitis C shared hopes that “curing” their disease would result in a reduction of stigma in their lives and a return to normality, some patients identified an “indivisible flow between pre‐cure and post‐cure life.”^22^ Patients’ medical records still indicated a history of hepatitis C, which invited assumptions regarding their past or present drug use. Thus, clinicians’ rhetoric and mannerisms toward them did not change, causing patients to experience the same enacted stigma and health care‐based discrimination in their lives after being cured. It is not far‐fetched to conclude that those “cured” of SCD will similarly continue to meet with stigma and health care‐based discrimination, as people with SCD are also commonly presumed to have a history of drug abuse.

## Social Suffering and Identity Change

At times, being “cured” of a condition can cause an identity crisis, an outcome that patients may not have factored into their understanding of cure and transformative therapy. For example, being “cured” of SCD can lead to social suffering as a patient is liberated from their familiar and well‐recognized disease identity and thrust into an identity in which the shared experiences of pain and stigmatization that define the SCD community are harder to recognize. Such a liberation is not necessarily a painful transition. For example, gene‐therapy recipient Ana Disla refers to the day that her sickled blood was transformed as her “rebirth day.”^23^ For Disla, gone was a life constantly interrupted by sickle crises, and receiving gene therapy was the start of a life in which she could achieve her dream of starting a clothing business.

Yet, for some, the transition can be painful and isolating. In learning to accept their disease and the possibility that they can die young, a person can grow connected to their disease identity as a means of forming a stable sense of self.^24^ Liberation from an inherited SCD identity can mean losing a familiar identity and replacing it with an identity that has never been known: an identity without SCD. Some may be unsure of how to live life as a “normal” or “healthy” person and may not know how to reenter the world without factoring SCD into their every decision. Some considering gene therapy may also worry that, without SCD, they will no longer be a recognized as a member of the “sickle cell warriors” community.^25^ “Sickle cell warriors” refers to those who live with SCD and perceive themselves as fighters in the daily battle against pain, discrimination, and other SCD‐related challenges. Some community organizations, sickle cell advocates, and others living with the disease have rallied around the identity of a sickle cell warrior.

The sense of identity that can accompany disease is readily recognized in cancer treatment. Although some people who have been treated for cancer may reject the identity of a “cancer survivor,” this term is used by many health care practitioners, researchers, and patients to refer to the impact of cancer on one's life for the remainder of one's life, even after the cancer has been eliminated.^26^ “Cancer survivor” can allow for a patient to acknowledge that their cancer diagnosis is still a part of their sense of self.

## Reproductive Concerns for Sickle Cell Persist

When gene‐editing therapy is described with curative language, patients may also assume that a “cure” includes the restoration of “normal” reproductive capacity—and this may very well not be the case. Given that patients with SCD generally experience shorter‐than‐average lives, reproductive health issues, family planning, and SCD have rarely been integrated into a SCD patient's wraparound experience of care.^27^ However, thanks to advances in screening and treatments and to transformative therapies like gene editing, patients with SCD are now expected to live longer and can embrace options to reproduce.

Yet chemotherapy is required for all SCD curative options—bone marrow transplant, stem cell transplant, and gene‐editing therapy—and it poses infertility risks, even sterilization, placing patients in the position to choose between one of these treatments and fertility preservation.^28^ Aware of the damage that chemotherapy can pose to the reproductive system, the American Society of Clinical Oncology released clinical guidelines for advising oncology patients on fertility‐preservation options,^29^ and fifteen states have introduced legislation that mandates insurance coverage of fertility preservation for oncology patients prior to chemotherapy and radiation treatment.^30^ In contrast to the situation for oncology patients, fertility preservation for SCD patients is not very accessible, and insurance costs for it are not clearly defined.^31^ SCD patients who are interested in gene‐editing procedures often must go to great lengths to secure access to fertility preservation.

Casgevy and Lyfgenia are somatic gene therapies, modifying the hemoglobin gene, but a fetus could still inherit the sickle cell trait from the mother, father, or both, regardless of the success of the gene‐editing treatment process for the patient. In other words, SCD patients who undergo treatments with Casgevy or Lyfgenia can still transfer the sickle cell trait to their offspring. Yes, these gene‐editing modifications can treat and modify the SCD patient's blood, but it needs to be clear that these therapies cannot alter the SCD patient's eggs, sperm, or reproductive cells. Patients who become pregnant may still face the question whether to engage in noninvasive prenatal testing for SCD screening in the fetus, and perspectives on using such prenatal tests to screen for SCD using cell‐free DNA in maternal plasma have been mixed.^32^ In vitro fertilization (IVF) combined with preimplantation embryonic selection may be a more desirable option for those hesitant to use noninvasive prenatal testing. The process of “freezing eggs” or becoming pregnant via IVF while navigating SCD, however, poses additional emotional and financial burdens. Limited scientific studies explore the combination of such dynamics among SCD patients, and thus there is little data on SCD patients who may desire these reproductive options.^33^

In the United States, the average cost of a pregnancy via IVF can range from $12,000 to $25,000, and the estimated cost can exceed roughly $61,000 when prenatal genetic testing for identifying embryos without sickle cell is included in totals.^34^ The combination of the costs for undergoing gene‐editing treatments plus IVF treatments creates high financial burdens for SCD patients, which can cause financial precarity. These are potential stressors that will not be eradicated for SCD patients imagining a “cure” for their disease. These genetic therapies may transform one aspect of the SCD patient's life, but when it comes to navigating reproductive health, the battle of SCD is ongoing.

The reality of reproductive health coupled with the lived experiences of SCD must be taken into account as a collective dynamic. This means understanding that the majority of SCD patients or parents who have children with SCD are generally at a greater financial disadvantage than oncology patients since the unemployment rate for those with SCD is as high as 60 percent and SCD greatly affects minoritized racial groups, who have a significantly increased risk of poverty.^35^ Families impacted by SCD are overall at a greater financial disadvantage with fewer comprehensive treatments than are patients with similar monogenic disorders, such as cystic fibrosis.^36^

In both reproductive health centers and SCD treatment centers, there are serious gaps in care for SDC patients due, in part, to limited medical training about the complex overlap between reproductive health and SCD. Inadequate care at the intersection of reproduction and SCD poses both physical and psychological harms to patients of reproductive age, considering the higher incidence of delayed menstrual cycles, low sperm counts, pregnancy loss, miscarriages, stillbirths, and maternal and infant deaths among people with SCD. A recent *Stat News* study investigated egregious cases of reproductive medical interventions for some SCD patients; of the fifty women with SCD who were interviewed, seven reported being coerced into sterilization. The researchers further uncovered that these harms were stimulated through obstetric racism and reproductive stratification.^37^ “Obstetric racism” refers in this context to detrimental forms of dismissive prenatal care, denial of pain, denial of informed consent, and additional forms of emotional, physical, and psychological neglect that violate Black women at disproportionately higher rates than white women.^38^ Scientific research on SCD patients’ experiences of reproductive health care and on their perceptions of reproductive decision‐making dynamics is under way, such as a forthcoming study on the increase of cesarean sections, obstetric racism, and coercive sterilization among Black women with and without SCD in the United States.^39^

## Well‐Balanced Approaches to Gene Therapy

We urge health care practitioners to be mindful of the language they use to describe gene‐editing therapy for SCD and to refrain from referring to it as the “cure” for the disease. Describing and discussing this intervention requires a well‐balanced approach that specifies the transformative features of gene‐editing therapy while acknowledging its limits with regards to the serious realities of SCD.

Avoiding curative language to describe gene‐editing therapy is not enough. To avoid implicitly framing gene‐editing therapy as a “cure,” hope must be provided with transparency about the kinds of physical pain and social hardships that may persist and about the reproductive risks and issues for SCD patients even if they undergo this therapy. Clinicians should also inform SCD patients of alternative treatments and of supportive services for the management of SCD, discussing which ones insurance might or might not cover. Not all patients with SCD will qualify for gene‐editing therapy, and for those who do qualify, the average $2.125 million bill may still be a barrier to accessing this option.^40^ Moreover, for the reasons we provided in this paper, eligible patients may ultimately refuse gene therapy, and these patients are still entitled to other SCD management options.

People with SCD have few pharmaceutical options to manage their disease compared to people with similar chronic and genetic disorders. Between 2008 and 2018, federal funding for research was greater per person with cystic fibrosis (CF) compared with SCD ($2,807 versus $812).^41^ Philanthropically supported research was also significantly greater per person with CF than per person with SCD ($7,690 versus $102), and the Food and Drug Administration approved four disease‐specific treatments for CF compared to one disease‐specific drug for SCD.^42^ Since 2012, three disease‐specific drugs that were approved for CF received five new indications, while hydroxyurea is the only SCD‐specific drug to receive a new indication (its use was finally approved for children in 2017). Hydroxyurea has been considered the gold standard for managing SCD, but some patients may have reservations about taking the drug because patients must stay on it indefinitely and it can increase the risk of fertility and pregnancy complications. In short, SCD has not received the attention it is due, and while new gene‐therapy treatment options will be beneficial for some patients, it is too soon to stop looking for improvements. To better serve SCD patients and their caregivers, the hopefulness surrounding these therapies must be met with radical honesty, starting with language that is representative of the lived experiences of SCD.

## Acknowledgments

We are grateful for the feedback from the audience at the American Society for Bioethics and Humanities annual meeting and from members of The Ohio State University's Center for Bioethics.
